# Paroxysmal Supraventricular Tachycardia and Cardiac Arrest: A Presentation of Pulmonary Embolism With Infarction as a Sequela of Long COVID Syndrome

**DOI:** 10.7759/cureus.18572

**Published:** 2021-10-07

**Authors:** Dhruv Talwar, Sunil Kumar, Sourya Acharya, Shivam Khanna, Vidyashree Hulkoti

**Affiliations:** 1 Department of Medicine, Jawaharlal Nehru Medical College, Datta Meghe Institute of Medical Sciences (Deemed to be University), Wardha, IND

**Keywords:** covid-19, paroxysmal supraventricular tachycardia, pulmonary embolism, pulmonary infraction, long covid

## Abstract

With the emergence of diverse post-COVID sequelae, there have been reports of thromboembolic events such as stroke, myocardial infarction, and pulmonary embolism. These events have been reported after severe coronavirus disease 2019 (COVID-19) infections mostly requiring intensive care unit admissions. The findings of acute pulmonary embolism on electrocardiography are commonly sinus tachycardia and S1Q3T3. However, the presentation of pulmonary embolism with arrhythmias is rare. We report a case of a young 31-year-old female who had a history of COVID-19 with a chest computed tomography (CT) severity score of 5/25 five weeks back and presented with acute onset chest pain, breathlessness for one hour followed by collapse. She was intubated in the emergency department and managed with antiarrhythmic drugs however she went into sudden cardiorespiratory arrest and was revived with cardiopulmonary resuscitation. The patient was finally diagnosed as a case of pulmonary embolism leading to pulmonary infarction presenting as paroxysmal supraventricular tachycardia and cardiac arrest as a result of long COVID syndrome. This emphasizes the importance of routine follow-up and strict vigilance even in young patients with mild COVID-19 as it might result in serious life-threatening complications which otherwise seem to be unexpected.

## Introduction

There has been an association of severe acute respiratory syndrome coronavirus 2 (SARS-CoV-2) with a procoagulant state leading to life-threatening events such as pulmonary embolism, myocardial infarction, and stroke. Abnormalities of various coagulation parameters were frequently encountered in COVID-19 patients and seem to be linked with poor prognosis. This procoagulant state is often associated with severe COVID-19 infection requiring intensive care unit admissions. Unfortunately, there is very little understood about COVID-19 associated pulmonary embolism as there is a lack of large prospective research for the same. Understanding the association of COVID-19 and long COVID with pulmonary embolism is extremely crucial to prevent potentially fatal complications of the lethal ongoing pandemic. The use of prophylactic anticoagulation and its optimal duration, as well as dosage in COVID-19 and long COVID syndrome, is a topic with different opinions. Pulmonary embolism was indeed reported to occur in COVID-19 patients who are critically ill despite the use of thromboprophylaxis which questions the possibility of a higher dose of thromboprophylaxis in severely ill patients [1].

Long COVID has gained increasing importance with the sequelae of COVID-19 showing varied presentations after COVID-19 infection leading to a rollercoaster ride of ill health. Long COVID has been defined as ongoing symptoms of COVID-19 which persist beyond four weeks from the initial infection. Common complaints of patients in long COVID include dyspnea, cardiovascular abnormalities, cognitive and mental health abnormalities, and olfactory as well as gustatory dysfunction [2]. We report a case of a young female with mild COVID-19 infection and no comorbidities who developed an unexpected complication of long COVID in the form of cardiac arrest and paroxysmal supraventricular tachycardia. This arrhythmia was a presentation of pulmonary embolism along with lung infarct presenting with acute onset dyspnea and chest pain followed by collapse. Cardiac arrest can be explained as a consequence of pulmonary embolism which has been reported previously in the literature [3]. While severe COVID-19-induced pulmonary embolism has been reported previously, our case report remains the first case report in the world to report mild COVID-19 with chest computed tomography (CT) severity score of 5/25 leading to nearly fatal pulmonary embolism and lung infarction leading to cardiac arrest as a sequela of long COVID, to the best of our knowledge.

## Case presentation

**Figure 1:**
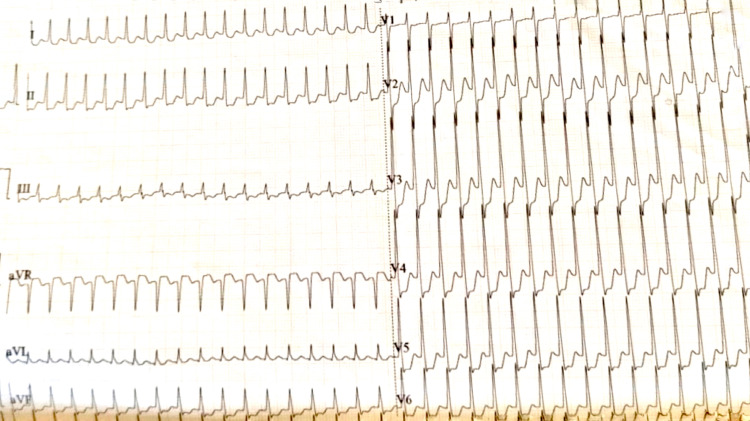
ECG of the patient showing paroxysmal supra-ventricular tachycardia

A 31-year-old female presented to the emergency department in an unresponsive state with the chief complaint of dyspnea and chest pain which was left-sided and sharp in nature for two hours followed by collapse. She had a history of mild dry cough for the past five weeks ever since she contracted COVID-19 when her CT severity score was 5/25, all her investigations including D-Dimer, C-reactive protein, and serum ferritin were within normal limits and she was home quarantined for the same. She had no history of fever, loss of taste or smell. There was no history of diabetes mellitus, hypertension, thyroid disorder, or any other co-morbidities. The patient was a nonsmoker. There was no history of supraventricular tachycardia or any other arrhythmias in the past. The patient was immediately intubated in view of airway protection. ECG was suggestive of paroxysmal supraventricular tachycardia with a heart rate of 150 beats/minute and diffuse significant ST depressions with ST elevation in the augmented vector right (aVR) (Figure 1).

She was managed with intravenous 6 mg adenosine. Cardiac markers were also measured revealing creatine kinase myocardial band (CK-MB) to be 10 IU/L and Troponin I 0.02 ng/mL. The patient went into sudden cardiorespiratory arrest and was revived with two cycles of cardiopulmonary resuscitation along with norepinephrine showing sinus rhythm on ECG. She was shifted to the intensive care unit. On general examination her pulse was 98 beats/minute, blood pressure was 90/60 mmHg in right arm supine position, oxygen saturation (SpO_2_) was 93 percent on T-piece with the oxygen of 4 liter and respiratory rate 28 breaths per minute. On systemic examination air entry was reduced on the left infra scapular area, heart sounds were normal, the abdomen was soft and non-tender and the patient was drowsy with bilateral plantar flexor and neuro deficit. Lab investigations revealed a raised D-Dimer level at 1.51 and the rest of the investigations are shown in Table 1.

**Table 1:** Lab investigation results of the case

Lab Parameter	Measured Value
Haemoglobin	11.9 gm/dL
Mean Corpuscular Volume	85 fL
Platelet Count	150000/dL
White Blood Cell Count	6400/dL
Total Protein	Total Protein-7.0gm/dL
Aspartate aminotransferase	22 units/L
Alanine aminotransferase	25 units/L
Alkaline Phosphatase	102 IU/L
Creatinine	0.9 mg/dL
Urea	24 mg/dL
C-Reactive Protein	10.0 mg/dL
D-Dimer	1.51
Serum Ferritin	330 ng/mL

**Figure 2:**
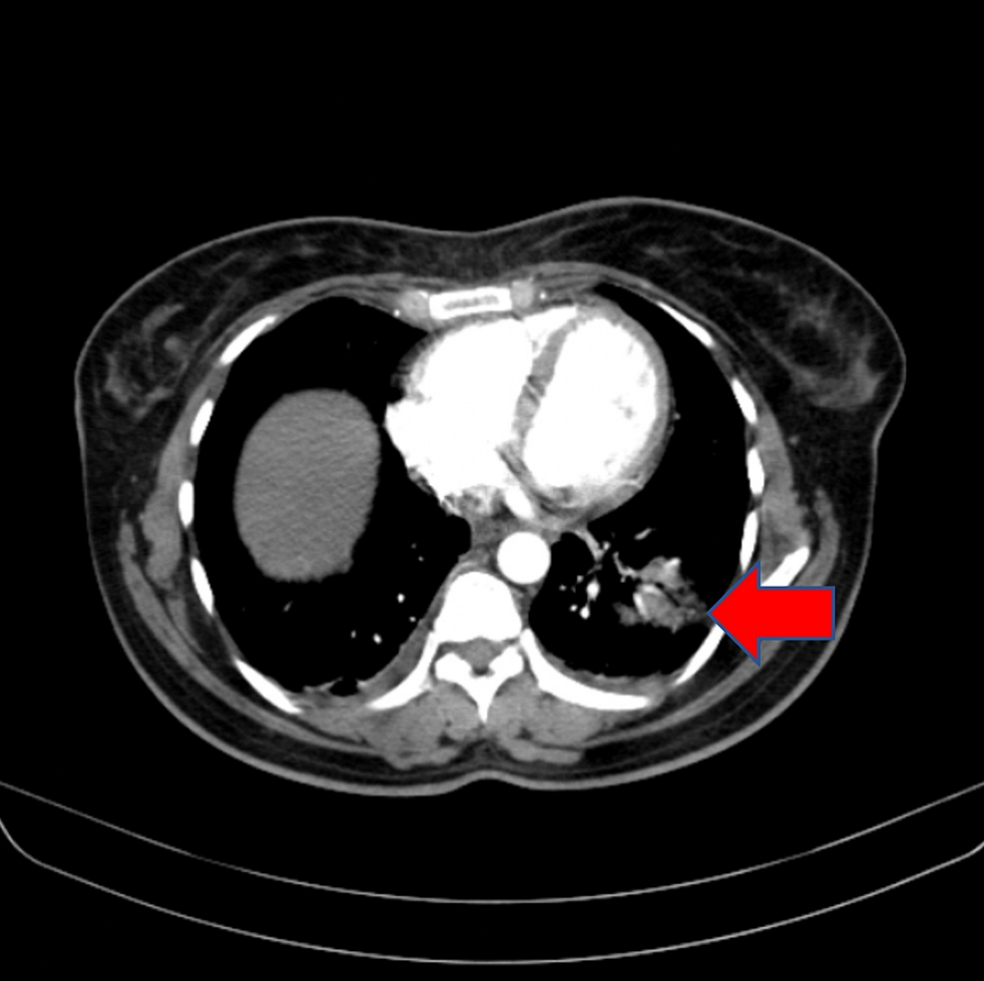
CT pulmonary angiography showing pulmonary infarction CT-Computed tomography

**Figure 3:**
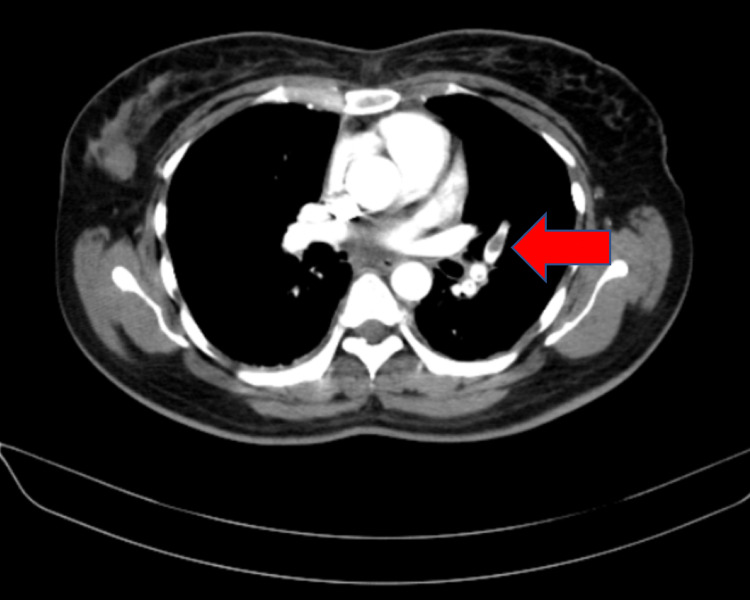
CT pulmonary angiography showing pulmonary embolism CT-Computed tomography

The patient was taken for computed tomography (CT) pulmonary angiography which showed pulmonary embolism along with pulmonary infarct as shown in Figures 2, 3. She was started on intravenous heparin 5000 IU six-hourly along with steroids. and other supportive measures. During the course of the hospital stay the patient improved clinically and she regained full consciousness and was extubated on day three of admission. Echocardiography was done which revealed no signs of wall motion abnormality or hypokinesia. Incentive spirometry and respiratory physiotherapy were given regularly. She was shifted from intravenous heparin to oral rivaroxaban 10mg BD (twice a day) on day four of admission. The patient was discharged in stable condition on day fifteen of admission and is currently doing well on follow-up. 

## Discussion

**Figure 4:**
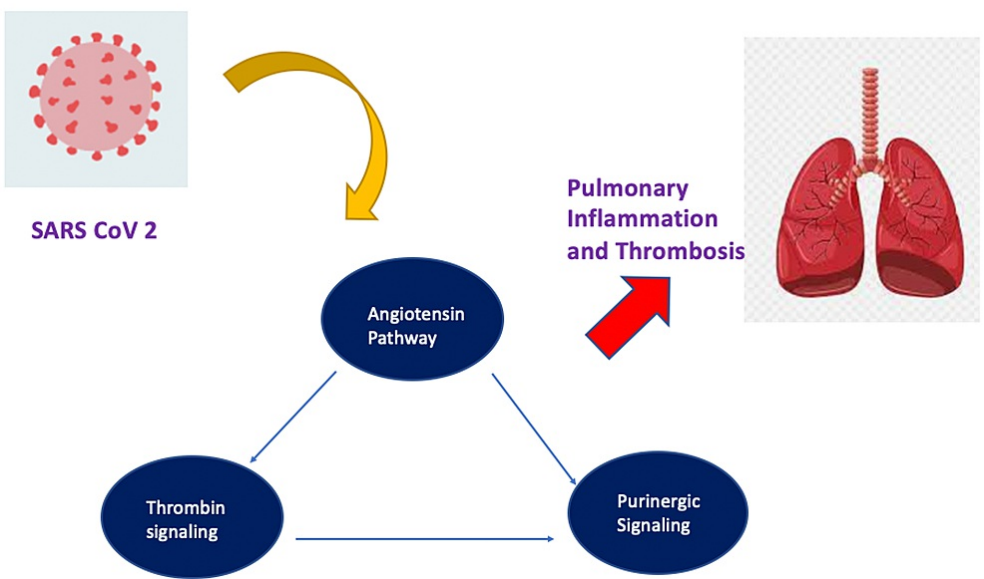
Pathophysiology of thrombosis in long COVID syndrome

An important cause of long COVID with acute onset dyspnea is pulmonary vascular thromboembolism [4]. Inflammation along with extensive tissue injury can occur as a result of dysregulated angiotensin signaling caused by SARS-CoV-2 binding to angiotensin convertase enzyme 2 [5]. This leads to an increase in the Tissue Factor production from the cells inside and near the alveoli [6]. The extrinsic coagulation cascade is therefore activated causing thrombin formation and signaling which exerts an effect on platelets as well as alveolar cells [7]. With the continuation of tissue inflammation and injury-causing thrombin-promoted platelet activation, there is enhancement activation of Factor XII and the intrinsic cascade of coagulation [8]. As the systemic inflammation increases in the severe cases of COVID-19, there is an increase in thrombin and purinergic signaling along with megakaryocytopoiesis, and platelet number may rise further leading to the promotion of thromboinflammation leading to a thromboembolic event such as pulmonary embolism as shown in Figure 4. Acute onset chest pain with dyspnea along with a history of COVID-19 was found along with prothrombotic state and an elevated D-dimer at presentation. This pointed towards pulmonary embolism as the likely cause of acute onset dyspnea, paroxysmal supraventricular tachycardia, and cardiac arrest in our case which was finally confirmed on a CT pulmonary angiography. The patient was managed conservatively with intravenous heparin and she responded well with medical management and physiotherapy.

Pulmonary embolism is usually seen with severe COVID-19 making our case an unusual one as our patient had a CT severity score of 5/25 and her inflammatory markers were normal when she had contracted COVID-19. She had a persistent cough after COVID-19 which she had neglected until she got acute onset dyspnea and chest pain. This might indicate a late-onset inflammatory response to SARS-CoV-2 which was missed due to no follow-up and lack of repeat blood investigations including inflammatory markers. Another unusual aspect of our case remains the presentation of pulmonary embolism as paroxysmal supraventricular tachycardia and cardiac arrest. Atrial tachyarrhythmias are uncommon presentations of pulmonary embolism. The probable explanation of atrial tachyarrhythmia in pulmonary embolism is the atrial stretch occurring from elevated right heart pressure from a pulmonary embolism [9]. Cardiac arrest as a result of pulmonary embolism can be caused due to pulmonary mainstream obstruction and liberation of various vasoconstrictive mediators from the thrombi, which leads to increased right ventricular afterload. As the right ventricle fails there is a rise in the pressure of the right atrium and cardiogenic shock occurs. The overload of the right ventricle also results in a leftward shift of the ventricular septum which leads to decreased left ventricular diastolic filling and end-diastolic volume. Ultimately there is a circulatory failure that occurs as a result of the decrease in left ventricular preload [10]. Thus, regular follow-up is essential even in mild COVID-19 to look for subclinical inflammation which might lead to potentially fatal complications such as arrhythmias inducing cardiac arrest pointing towards an underlying lung infarction due to long COVID syndrome. 

## Conclusions

We conclude that pulmonary embolism with pulmonary infarction is a rare but important complication of mild COVID-19-induced long COVID syndrome with a rather uncommon presentation in the form of paroxysmal supraventricular tachycardia and cardiac arrest. It is therefore essential for patients to have a regular follow-up after contracting COVID-19 to prevent potentially life-threatening complications which may arise with long COVID. Also, the clinicians should be aware of atypical presenting features of pulmonary embolism and infarction in the form of arrhythmias which need prompt diagnosis and management, such as in our case, to prevent mortality.
